# The Relation between Dental Caries and Diet

**Published:** 1891-07

**Authors:** L. Ashley Faught

**Affiliations:** Philadelphia


### THE RELATION BETWEEN DENTAL CARIES AND DIET.[1]

BY L. ASHLEY FAUGHT, D.D.S., PHILADELPHIA.

A PERFECT understanding of the relation we are about to consider must necessarily lie somewhat in a comprehension of the term "dental caries." Many definitions have been proposed, and each with its weight of authority; but I would express the following as possibly the one of most general acceptation: Dental caries is purely chemical alteration of the enamel and ivory of the teeth. This alteration is in its final results destructive of the tissues involved, and progresses from the outside to the inside of the tooth. The peculiar province of the dentist is threefold,—to arrest this action, to repair its ravages, and to defend the teeth from its attacks. The last mentioned is in its nature essentially preventive, and is to be comprehended largely by a study of the relations of diet. I admit that the effects of diet are also reparative; but I conceive a special interest in its bearings as a protective agency, and to this I desire now to invite your attention.

As a preventive means, diet either exerts a well-marked influence upon the anatomical structure of the teeth, or it has for its aim the suppression of contact with teeth of agents of alteration, or the neutralization of their powers. The influence upon anatomical structure is confined to the tissues other than enamel, for this substance is not reconstructed, consisting as it does of a single series of cells, and, being once lost, is lost forever. Such influence also requires necessary conditions under which to operate, proper pabulum, proper state of the organism to appropriate, and proper power to assimilate. That the system rejects all chemical foods from the chemist's laboratory, and only receives things which come in nature's own way, is a maxim so true that it is worthy to be laid down as a fact in connection with this phase of the study. Phosphate of lime is a tooth-essential, but nature uses only those phosphates which are presented to her in connection with albumen. Wheat is a source of its supply, par excellence. Oatmeal, which has phosphate of lime so necessary for infants, should be mixed with cow's milk when giving the latter in the place of mother's milk. The age of childhood is particularly the age for the structural effects of appropriate

[1] Read before the Pennsylvania State Dental Society, June, 1890.

feeding, for functional action is not the same in all ages, and this makes a great difference in the power of repair, which seems to become lost at later periods.

While one cannot doubt that it is possible to influence nature by appropriate diet, yet the fact that but little repair in more mature human teeth has been discovered, though greatly needed, would indicate that she has not the ability to do it. Any study, therefore, of the relation of diet to dental caries will prove profitable, not so much in proportion to the effort expended in an examination of its effects upon the *inside* of tooth-structure, as in the comprehension of the bearings productive of *outside* elements which may act upon the teeth.

Recognizing, then, this fact, the point of our observation is to be directed to a consideration of the chemical changes which diet may produce in the fluids of the mouth; and which by their perverted condition destroy the enamel, and lead up to those secondary actions, which, being permitted entrance through this external plate, run riot in the softer structures, breaking down and destroying the teeth. The normal saliva being alkaline, must through such agencies become acid. These acids here factoring are either sulphuric, hydrochloric, nitric, or lactic. They are produced more frequently through the direct relation of the food, though not infrequently through derangement in the intestinal tract. This latter condition is to be met by guarding against irregular eating, for trouble will surely follow eating between meals, a habit which seems to be firmly engrafted upon the present generation.

Children, following the bent of their own inclinations, and led on by the prejudicial example of their elders, fail to perceive that the stomach and its appendages are simply a chemical laboratory; and that material introduced at any one period requires a definite time for proper disposal; and that the retorts, tubes, and chemicals used in the process need time for repair and replenishment. If this quantum of rest is not given, they will surely be in bad condition and do very imperfect work. This fact should in this age be taught with emphasis until our *clientèle* accept the delicate adjustment of the human organism, and learn that indigestion is an immense factor in the disturbance of perfect nervous balance, and that lowered nervous tone means a proportionate lowered tone in tooth-structure. Here, then, is a cause in dental caries which should not be forgotten in answering the oft-repeated question, presented to us with much anxiety of mind,—Why do my teeth so decay?

One should know, too, that ruts are not always good things in which to fall, and carefully avoid sameness of food. Change in diet calls for change in its chemical disposal, and evil effects are not continued long enough to leave their impress upon the dental organs; while variety will always continue a full supply of the necessary ingredients of tooth-tissue, for food quite different in the form of its presentation is nevertheless rich in nearly all instances in the same elements out of which nature by recombination may produce continually a definite structure.

Let us now consider the production of acids in the mouth. Recognizing the presence of nitric acid in the white decay around fillings and the necks of the teeth, the food recommended should be rich in carbon and not in nitrogen. Non-nitrogenous food is indicated,—lean meats, eggs, cheese, etc., are to be avoided. When the acid reaction is due to hydrochloric acid, the patient may partake freely of vegetables and milk, but not at all of salt foods. The presence of black decay, due to sulphuric acid, should debar food containing sulphur; while evidences of erosion indicating the action of lactic acid must in like manner promptly erase from the diet-list such articles as milk, meats, saccharine materials, and amylaceous substances which are so easily converted into dextrine.

The two points which I desire to recapitulate as the lesson for concentrated consideration are, that dental caries requires the outer tooth-plate to be broken, disintegrated, or removed before further progress can take place, and this destruction is in a large measure due to the action of acid oral fluids; and that whether the oral fluids shall be acid or alkaline is largely dependent primarily upon the condition of the digestive system, or secondarily through its reflex disturbance of the nervous system. Keep the digestive system normal, and the nervous system in serene equilibrium, and, all things being equal, a limit is placed upon dental caries.

I trust that the province of this paper may not be misunderstood, and I do not wish to be assigned a place among those who do not comprehend or appreciate the other accepted causes of dental caries, such as germ potency, lowered vitality, vicious approximation, heredity, etc. They are factors to be recognized, but the truth as here enunciated is to my mind common-sense, solid doctrine, and will prove a sound basis upon which may be founded a system of advice to patients productive of much salvation in tooth-structure.

